# Knowledge and Attitude Toward Child Safety Seat Use in Saudi Arabia: A Cross-Sectional Study

**DOI:** 10.7759/cureus.54813

**Published:** 2024-02-24

**Authors:** Hanan Mashi, Esra Alamri, Shahd Alotaibi, Jamal A Omer

**Affiliations:** 1 Pediatrics, King Fahad Central Hospital, Ministry of Health, Jazan, SAU; 2 Pediatrics, Children Specialized Hospital, King Fahad Medical City, Riyadh Second Health Cluster, Ministry of Health, Riyadh, SAU; 3 Clinical Research, King Fahad Medical City, Riyadh, SAU; 4 Pediatrics, King Fahad Medical City, Central Second Health Cluster, Ministry of Health, Riyadh, SAU

**Keywords:** child behavior, road traffic accidents, attitudes, knowledge score, child safety seat

## Abstract

Background

Car safety seats (CSS) are a well-established strategy to reduce injuries and fatalities among children involved in road traffic accidents. However, the use of CSS is generally low globally due to limited knowledge of its benefits. This study assessed parents' knowledge and attitudes toward CSS in Saudi Arabia.

Methods

This cross-sectional study used an online self-administered survey distributed among residents in different regions of Saudi Arabia to assess their knowledge and attitude toward CSS. Data were analyzed using the statistical package for the social sciences (SPSS) version 23.0 (IBM Corp., Armonk, NY).

Results

A total of 383 Saudi residents participated in the study. The mean age was 37.14 ±9.10 years, with a female predominance (62.1%, n=238). One-third of the participants were from the western region of Saudi Arabia (30.3%, n=109). Non-use of CSS was reported by 25.8% of the participants, while 11.7% reported that they rarely used CSS. The mean total knowledge score was 2.15 (range 0 -3). A lower knowledge score was significantly associated with a lower educational level (p=0.008), not having information regarding CSS (p=0.005), none or rare use of CSS by the parent (p<0.001), and the use of media or self-education as a source of information regarding CSS (p=0.002). The mean attitude score was 12.52 (range 4 -20). The attitude score was significantly associated with gender (p=0.002), education (p=0.014), number of children (p=0.005), monthly family income (p=0.028), use of CSS by people other than the parent (p<0.001), information about CSS use in the car (p<0.002), source of information (p<0.001), and use of CSS by the parent (p<0.001).

Conclusions

The knowledge and attitude of the Suadi population toward CSS use are inadequate, highlighting the need to enhance awareness and understanding of the importance of CSS use.

## Introduction

Road traffic accidents (RTAs) pose a significant public health issue and are a leading cause of mortality and morbidity worldwide. Globally, around 1.2 million individuals die annually in road crashes, and 50 million suffer severe injuries [1]. The RTAs in Saudi Arabia pose a major threat to public health, causing injuries and fatalities. According to the Ministry of Interior (MoI), in 2019, there were 287,781 RTAs, leading to 5,754 deaths, adversely impacting public health and economic status [2].

Studies have consistently shown that fatalities among children in road crashes are often caused by their ejection from the vehicle during the collision [3]. To prevent these fatalities, it is crucial to properly secure children in vehicles using proper restraints [4]. Car safety seats (CSS) are recommended for children in vehicles based on the child's age, weight, and vehicle type. There are various types of CSSs available, including rear-facing seats for infants, convertible seats for children up to six years, forward-facing seats for children nine months to 11 years, booster seats for children four to 11 years, and wedge pillows for children six to 11 years depending on their weight [5]. The current body of evidence shows the advantages of utilizing CSS in vehicles. Previous studies have shown that using an age, stature, weight, and physical limitation-appropriate restraint can significantly reduce childhood mortality by 50%-75% and life-threatening injuries in infants and toddlers by 71% and 54%, respectively [6,7].

Despite their well-established benefits, only 33 out of 84 countries implementing CSS laws meet the best practices criteria set by the World Health Organisation (WHO) [8]. Additionally, studies showed low usage of CSS in several countries; barriers such as inconvenience, cost, child behavior, discomfort, and a negative parental attitude toward their importance were linked to the low utilization of CSS [9,10]. The current evidence highlights poor knowledge and unfavorable attitudes among the public concerning CSS practice. For instance, a study conducted in Nigeria revealed that 26% of parents surveyed considered CSSs unnecessary [9]. Similar reports from Turkey [11], South Africa [12], Singapore [10], and Romania [13]. Studies have also found that a lack of knowledge regarding the proper use of CSS is a barrier to their usage. In 2018, a study in Athens revealed that 38.5% of the participants never received education on the use of CSS [14].

In Saudi Arabia, CSS use is regulated by the government, which has effectively promoted its usage; however, there are no national guidelines or enforcement for appropriate CSS use [15]. According to recent laws, all children up to four years in Saudi Arabia must be provided with CSS. Nearly two-thirds of the Saudi population expressed that children do not necessarily need to be seated in CSS [16]. Local studies from Saudi Arabia showed that 28% of participants held the same perspective [17]. Another Saudi study demonstrated that 64.3% of participants showed poor knowledge of CSS use, and 64.0% were practicing improper CSS use [18]. While previous studies have addressed the topic of the road safety of children in Saudi Arabia, these studies were limited to specific regions only, and data at the national level are lacking. Thus, this study aimed to assess the knowledge, attitude, and practice of using CSS among parents in Saudi Arabia.

## Materials and methods

Study design and population

A cross-sectional study was conducted among Saudi Arabian residents, targeting a diverse range of parents, including citizens and residents of all ages. The study utilized an online self-administered questionnaire distributed through various regions of Saudi Arabia through social media platforms -including Facebook and X (previously Twitter), among others - between September 2020 and April 2021. We targeted parents over 18 years old with at least one child under 12 years old at the time of data collection. To ensure that we reached the intended audience, we employed targeted advertising and social media campaigns, focusing on online communities and forums frequented by parents. These included parenting groups on social media and online forums for family healthcare.

Sample size calculation and sampling technique

The sample size was determined using a margin of error of 5%, a confidence interval (CI) of 95%, and an expected response rate of 50% to most of the main questions. We estimated a minimum sample size of 384. The calculation was derived from the following formula: n=(Z^2 * p * (1-p))/E^2, where n is the sample size, Z is 1.96 for a 95% CI, p is the expected response rate of 0.50, and E is the margin of error of 0.05. We adopted a non-probability convenient sampling technique to recruit eligible residents.

Questionnaire development and validation

A self-administered, online-based questionnaire to assess the practice/use of CSS was developed (Appendix 1) by reviewing the available questionnaires in the literature [9,17,18]. The questionnaire was distributed in English language and consisted of three sections: demographic, knowledge, and attitude. The demographic section included questions on age, gender, the highest level of education, the number of children, region of residency, average age of children, and family income. The knowledge section had questions on the previous knowledge of relevant traffic laws and regulations on driving a car without CSS and the best place for installing and directing the CSS in the vehicle. The attitude section comprised four items concerned with the parents' stance on CSS. Parents were asked to give their attitude toward seating a child on an adult's lap and in the front seat and the need to use CSS for an eight-year-old and long-distance travel. The questionnaire also assessed the use of CSS among responders using a 4-point Likert scale question ranging from always to never. A scoring system was developed to score knowledge and attitude. Each knowledge question was scored 1 for correct/appropriate answers and 0 for incorrect/inappropriate answers, with a total knowledge score ranging from 0 to 3. Responses with a total knowledge score of 3 were categorized as having good knowledge. A 5-point Likert scale was used to score the attitude toward CSS use, ranging from strongly agree (score of 5) to strongly disagree (score of 1); the total attitude score ranged from 4 to 20, and responses with an overall score of 16 were categorized as favorable attitude.

Before data collection, the questionnaire underwent a rigorous pilot testing phase to ensure its reliability and validity. This preliminary phase involved administering the questionnaire to a small, representative subset of the target population. Feedback regarding the clarity, relevance, and comprehensiveness of the questions was collected and analyzed. Based on this feedback, adjustments were made to refine the wording, order, and format of the questions. In addition, two subject matter experts were consulted to assess content validity, ensuring that the questions adequately covered the key aspects of the CCS knowledge and attitude. For reliability testing, we calculated the internal consistency of the questionnaire using Cronbach's alpha; the results showed a Cronbach's alpha of 0.629. This value indicates a moderate level of internal consistency among the items in the questionnaire.

Statistical analysis

Data were analyzed using Statistical Package for Social Studies (SPSS 22; IBM Corp., Armonk, NY, USA). Continuous variables were expressed as mean ± standard deviation and categorical variables as percentages. The Shapiro-Wilk test was used to assess the normality distribution for the variables. Kruskal-Wallis and Mann-Whitney U tests were used for continuous non-parametric variables, while the Chi-square test was used for categorical variables. An odds ratio (OR) with a 95% CI was used to assess the associated factors with low knowledge and unfavorable attitudes. A p-value of <0.05 was considered statistically significant.

## Results

Demographics

The study received 383 complete responses. The mean age of the participants was 37.14 ±9.10 years, with a female predominance (62.1%). Participants were distributed across the regions of Saudi Arabia, with a slightly higher percentage in the Western region (30.3%, n=109), followed by the Central region (27.5%, n=99). Regarding the education level, 75.2% of the population had a university degree or higher education. Nearly half of the sample had one or two children (48.3%, n=180), and 43.6% (n=167) had children older than eight years. Additionally, 60.3% of the sample (n=231) reported an average monthly family income of more than 2,700 USD (Table 1).

**Table 1:** Demographic characteristics of the participants

Variables		Responses (N =383)	
		Number	%
Age	Mean ±SD	37.14 ±9.10	
Female sex		238	62.1
Region	Southern area	88	24.4
	Western area	109	30.3
	Eastern area	45	12.5
	Northern area	19	5.3
	Central area	99	27.5
Education	Primary/secondary	17	4.4
	High School/Diploma	78	20.4
	University education or higher	288	75.2
Number of Children	1-2	180	48.3
	2-5	144	38.6
	>5	49	13.1
Average age of children (years)	<2	103	26.9
	2-4	138	36.0
	5-8	142	37.1
	>8	167	43.6
Monthly family income (USD)	<1080	29	7.6
	1080-1890	31	8.1
	1890-2700	92	24.0
	>2700	231	60.3

Characteristics of the parents

Nearly 26% of the sample reported that one more person was responsible for transporting the children. Participants with prior information on CSS comprised 87.2% (n=334) of the sample. The media was the commonest source of information for more than half of the participants (59%, n=197), followed by self-education (31.1%, n=104). Almost 98% (n=374) of the participants emphasised the need to increase the community's awareness regarding the importance of CSS. A quarter of the sample indicated non-use of CSS (25.8%, n=99), while 36, 26.4, and 11.7% stated that they always, sometimes, and rarely used safety seats, respectively. Regarding the reasons for the non-use of CSS, 42 (42.4%) participants cited insufficient space, 32 (32.3%) cited high cost, and 30 (30.3%) participants considered it unnecessary (Table 2).

**Table 2:** Characteristic of the parents

Variables		Responses (N =383)	
		Number	%
Does someone other than the parents deliver the child?	No	284	74.2
	Yes	99	25.8
Do they use car safety seats?	No	142	37.1
	Yes	150	39.2
	Sometimes	91	23.8
Do you have information about safety seats in the car	No	49	12.8
	Yes	334	87.2
If yes, the source of information	Other	12	3.6
	Media	197	59.0
	HCWs advice	21	6.3
	Self-Education	104	31.1
Do you know the traffic laws and regulations related to driving a car without a safety seat?	No	153	39.9
	Yes	230	60.1
Do you think we need more community awareness regarding the use of child safety seats inside the car?	No	9	2.3
	Yes	374	97.7
Do you (the driver) use a seat belt while driving a car?	No	13	3.4
	Yes	370	96.6
Have you ever used a child safety seat inside the car?	Never	99	25.8
	Rarely	45	11.7
	Sometimes	101	26.4
	Always	138	36.0
If she does not use the safety seat, please select the reason (There is not enough space)	No	57	57.6
	Yes	42	42.4
If she does not use the safety seat, please select the reason (The prices are expensive)	No	67	67.7
	Yes	32	32.3
If she does not use the safety seat, please select the reason (I don't think it's necessary)	No	69	69.7
	Yes	30	30.3
If she does not use the safety seat, please select the reason (Other)	No	90	90.9
	Yes	9	9.1

Knowledge regarding CSS

Table 3 displays the responses to the knowledge and attitude domains. Most participants (95.8%, n=367) knew correctly about the ideal location for a child to sit in a car. In comparison, a nearly equal number of participants demonstrated an accurate understanding of the traffic regulations related to driving a vehicle without using a safety seat (60.1%, n=230) and the proper orientation of the child safety seat in the car (59%, n=226). The mean knowledge score was 2.15 (range 0 -3).

**Table 3:** Responses to knowledge and attitude questions

Variables	Responses (N =383)
Knowledge subscale	
Do you know what the traffic laws and regulations are related to driving a car without using a safety seat?, No. (%)	230 (60.1)
What is the best place for the child to sit in the car, No. (%)	367 (95.8)
Whichever direction is the child safety seat facing in the car?, No. (%)	226 (59.0)
Total score of knowledge, mean (range)	2.15 (0 -3)
Attitude subscale, mean (range)	
I think having an adult carrying a child in the car eliminates the need for a safety seat	3.37 (1-5)
Having the child in the front seat is appropriate when using a seat belt	3.76 (1-5)
Children eight years of age and older do not need a safety seat if the seat belt is properly secured	1.95 (1-5)
A safety seat is only important when travelling long distances or driving at high speed	3.44 (1-5)
Total score of the attitude	12.52 (4 -20)

The overall attitude score was 12.52±3.43. The highest mean score (3.76±1.17) was concerning the perception of having the child in the front seat as appropriate when using a seat belt, while the lowest mean score (1.95±0.91) was regarding the agreement that children eight years of age and older do not need a safety seat if the seat belt is properly secured.

Table 4 presents the association of knowledge and attitude scores with demographic factors. Participants with higher education levels had higher knowledge scores (p=0.008). Moreover, using CSS by people other than parents, having information regarding CSS, the source of information regarding CSS, and use of CSS by the parent was found to be significantly related to knowledge score, p-values being <0.001, 0.005, 0.002, and <0.001, respectively.

**Table 4:** Association between demographic factors and knowledge and attitude scores * Significant p-value # out of 3 % out of 20

		Knowledge^#^			Attitude^%^		
		Mean	SD	P value	Mean	SD	P value
Age	17-30 years	2.22	0.70	0.115	12.66	3.65	0.195
	31-40 years	2.20	0.72		12.79	3.21	
	41-50 years	2.05	0.75		12.12	3.59	
	51 years and above	1.87	0.96		11.65	3.31	
Gender	Female	2.19	0.75	0.167	12.10	3.41	0.002*
	Male	2.08	0.74		13.21	3.38	
Region	Southern area	2.20	0.66	0.776	12.34	3.58	0.372
	Western area	2.16	0.80		12.62	3.45	
	Eastern area	2.11	0.71		12.09	3.55	
	Northern area	2.00	0.82		12.00	2.19	
	Central area	2.20	0.78		13.25	3.44	
Education	Primary/Secondary	1.59	0.80	0.008*	10.71	3.55	0.014*
	High School/Diploma	2.18	0.77		11.91	3.33	
	University or higher	2.17	0.73		12.79	3.41	
Number of Children	1-2	2.13	0.76	0.864	13.00	3.44	0.005*
	2-5	2.17	0.73		12.24	3.29	
	>5	2.16	0.80		11.31	3.68	
Monthly family income (USD)	<1080	1.83	0.85	0.067	10.66	3.96	0.028*
	1080-1890	2.03	0.84		12.10	3.74	
	1890-2700	2.26	0.72		12.77	3.08	
	>2700	2.16	0.73		12.71	3.40	
Do people other than the parents use car safety seats?	No	1.89	0.76	<0.001*	11.13	3.20	<0.001*
	Yes	2.31	0.67		14.24	3.01	
	Sometimes	2.27	0.76		11.85	3.25	
Do you have information about safety seats in the car	No	1.88	0.73	0.005*	10.86	3.77	<0.002*
	Yes	2.19	0.75		12.76	3.32	
Source of information	Other	2.33	0.78	0.002*	13.33	2.96	<0.001*
	Media	2.06	0.79		11.78	3.17	
	HCWs advice	2.62	0.50		13.67	2.63	
	Self-education	2.33	0.63		14.38	3.09	
Do you think we need more community awareness regarding the use of child safety seats inside the car?	No	2.22	0.67	0.832	10.11	5.11	0.196
	Yes	2.15	0.75		12.58	3.37	
Have you ever used a child safety seat inside the car?	Never	1.84	0.80	<0.001*	10.58	3.19	<0.001*
	Rarely	1.91	0.76		11.44	3.55	
	Sometimes	2.27	0.76		12.29	2.77	
	Always	2.36	0.59		14.43	3.01	
* Significant p-value							
# out of 3							
% out of 20							

The participants' attitude was found to be significantly related to gender, with females scoring a higher mean attitude score than males (p=0.002) and education level, with the lowest mean attitude score among those with primary/secondary education (p=0.014). The number of children was also associated with the mean attitude score, where significantly higher scores were reported among those with >5 children (p=0.005). The attitude was significantly related to monthly family income, with the highest attitude scores seen among those with an income <1080 USD (p=0.028). The attitude was also significantly related to the use of CSS by people other than the parent (p<0.001), information about CSS use in the car (p<0.002), source of information (p<0.001), and use of CSS by the parent (p<0.001).

The logistic regression analysis showed a significant association between low attitude score and female gender (OR= 1.815, 95% CI: 1.195 to 2.756), participants with education level as high school/diploma (OR= 2.005, 95% CI: 1.182 to 2.432), those with 2-5 children (OR= 1.561, 95% CI: 1.001 to 2.432), participants who did not use CSS (OR= 7.133, 95% CI: 4.252 to 11.966), and media as a source of information (OR= 3.913, 95% CI: 2.366 to 6.470). Moreover, those who never, rarely, or sometimes used a CSS inside the car were significantly associated with a poor level of attitude (OR= 9.345, 95% CI: 5.070 to 17.226), (OR= 4.732, 95% CI: 2.305 to 9.715), and (OR= 3.761, 95% CI: 2.187 to 6.467) (Table 5).

**Table 5:** Factors associated with poor attitude * Significant p-value # Used as a reference

		Odds ratio	95 % CI	Upper	P value
			Lower		
Age	17-30 years	.543	0.234	1.262	0.156
	31-40 years	.559	0.248	1.261	0.161
	41-50 years	.608	0.255	1.453	0.263
	≥51 years^#^	1.00			
Gender	Female	1.815	1.195	2.756	0.005*
	Male^#^	1.00			
Region	Southern area	1.118	0.629	1.985	0.705
	Western area	1.249	0.724	2.156	0.424
	Eastern area	1.276	0.628	2.589	0.500
	Northern area	2.857	0.956	8.536	0.060
	Central area^#^	1.00			
Education	Primary/Secondary	1.734	0.625	4.815	0.291
	High School/Diploma	2.005	1.182	3.403	0.010*
	University or higher^#^	1.00			
Number of Children	1-2^#^	1.00			
	2-5	1.561	1.001	2.432	0.049*
	>5	1.761	0.919	3.374	0.088
Monthly family income	<4K	2.514	1.070	5.906	0.034
	5-7K	1.741	0.798	3.797	0.163
	7-10K	1.301	0.799	2.119	0.289
	>10K^#^	1.00			
Do they use car safety seats?	No	7.133	4.252	11.966	<0.001*
	Yes^#^	1.00			
Do you have information about safety seats in the car	No	1.786	0.947	3.369	0.073
	Yes^#^	1.00			
Source of information	Other	1.408	0.417	4.758	0.582
	Media	3.913	2.366	6.470	<0.001*
	HCWs advice	1.213	0.460	3.201	0.696
	Self-education^#^	1.00			
Do you think we need more community awareness regarding the use of child safety seats inside the car?	No	1.631	0.402	6.620	0.494
	Yes^#^	1.00			
Have you ever used a child safety seat inside the car?	Never	9.345	5.070	17.226	<0.001*
	Rarely	4.732	2.305	9.715	<0.001*
	Sometimes	3.761	2.187	6.467	<0.001*
	Always^#^	1.00			
* Significant p-value					
# Used as a reference					

## Discussion

This study assessed the knowledge and attitudes towards CSS use among Saudi Arabian parents. In the present study, the use of CSS was inadequate since non-use was reported by 25.8% of the sample, while only 36% reported always using it. The level of knowledge regarding CSS in Saudi Arabia was low, particularly concerning awareness regarding the traffic laws related to driving a car without using a CSS and the direction the CSS should face in the car. Only 60% of participants responded with the correct answer. Additionally, the Saudi population showed an unfavorable attitude toward the use of CSS. The results showed the respondents had particularly unfavorable attitudes toward using CSS in children eight years and older.

Our findings align with several previous reports showing inadequate knowledge levels and unfavorable attitudes toward CSS use. A previous study reported that even if CSS was being used, nearly 88.5% were misusing it [19], including positioning it in the wrong direction [20]. Likewise, a cross-sectional study of 350 parents from Unaizah City in Saudi Arabia showed low knowledge and an unfavorable attitude toward CSS use [18]. Another report showed that parents from Buraidah, Saudi Arabia, had a low level of utilization of CSS, and most respondents were unaware of CSS's importance and regulations in Saudi Arabia [21]. A more recent report from Jeddah, Saudi Arabia, recruited 192 mothers to assess their knowledge and practice of CSS. Nearly two-thirds of the mothers did not have a CSS in their cars and were unaware of the appropriate car-sitting methods [22]. A 2023 population-based study from Saudi Arabia reported that 65.3% of the Saudi population did not change their attitude towards using CSS, even after implementing the new laws mandating it. In addition, the level of knowledge of the importance of CSS was low [23].

The findings from the current study reveal that attitude towards CSS was related significantly to the participants' socio-demographic characteristics, including gender, education level, number of children, and monthly family income. The regression analysis showed that female gender, high school/diploma as the highest education level, and having two to five children were independent predictors of poor attitude towards CSS. Our findings are in line with the local study conducted in Unaizah, which found gender (p<0.001), educational level (p=0.004), and the number of children (p=0.009) to be significantly associated with attitude [24]. Other reports from Saudi Arabia showed similar findings [21-23]. Such results align with the need to address gender-specific concerns or misconceptions about CSS use. Additionally, there is a potential gap in awareness or understanding of CSS benefits among specific educational groups. Furthermore, the fact that having two to five children was an independent predictor of a poorer attitude could be attributed to practical challenges such as the cost or complexity of using multiple CSS or a perceived inconvenience. These insights are critical for tailoring effective educational and intervention strategies to improve attitudes and practices regarding child safety seat use in specific demographic groups.

Perceiving CSS as unnecessary was another reason for non-use, which may indicate low awareness regarding the importance of CSS. In this regard, it is worth mentioning that in the current study, media was the most common source of knowledge regarding CSS mentioned by more than half (59%) of the participants. Additionally, it was found that using healthcare workers as the source of knowledge was associated with significantly higher knowledge scores.

A notable finding of the present study is that perceiving CSS as unnecessary emerged as a reason for its non-use among participants. This perception likely stems from a low awareness or understanding of the importance and effectiveness of CSS in ensuring child safety. This aligns with previous research indicating that misconceptions and lack of awareness are significant barriers to adopting CSS practices [4]. Furthermore, the study showed that media was the most common source of CSS knowledge, cited by more than half (59%) of the participants. This indicates the media's substantial role in disseminating information about child safety. The high reliance on media for information underscores its potential as a tool for public health campaigns to improve CSS awareness and use. However, the quality and accuracy of the information disseminated through media channels can vary, which makes it crucial to ensure that the public receives correct and helpful advice. Recently, social media campaigns were found to be effective in increasing the awareness of CSS practices in Saudi Arabia [25], which warrants further investigation.

Economic factors, particularly the affordability and accessibility of child safety seats, are significant contributors to the use of CSS. Previous studies showed that common reasons mentioned for the non-use of CSS included insufficient space and high cost [11,26]. It was seen in South Africa, where the affordability of CSS caused financial burdens on families, especially those with more than one kid who needs CSS [12]. These findings have practical implications for policymakers to increase the use of CSS among the local population by reviewing the cost of CSS and encouraging more healthcare workers to educate parents regarding the importance of using CSS for their children [27]. Overwhelmingly, a recent report from Saudi Arabia showed that 92.8% of the respondents agreed that financial support would increase compliance with CSS use [23].

We recognize that our study is limited by being cross-sectional, meaning that we cannot establish causal relationships. Moreover, our study included all residents from different regions of Saudi Arabia but did not classify them as citizens or expatriates. Therefore, we recommend comparing the CSS use among these groups in future studies. It should be noted that this study was conducted before the new rulings on car safety for children in Saudi Arabia, as well as the tighter legislation and the fines that were imposed on parents [28]. Additionally, we calculated the internal consistency of the questionnaire using Cronbach's alpha; the results showed a Cronbach's alpha of 0.629. This value indicates a moderate level of internal consistency among the items in the questionnaire. While a Cronbach's alpha of 0.629 is below the commonly accepted threshold of 0.7 for good internal consistency, it is not uncommon for exploratory research or scales measuring diverse constructs. In our study, we employed a non-probability convenient sampling technique, which may not accurately reflect the broader population's characteristics due to the non-random selection of participants, leading to an over-representation or under-representation of certain groups.

Furthermore, in our study, the questionnaire was distributed in English, a decision influenced by the common use of English in professional and academic settings within Saudi Arabia. However, we recognize that English may not be the first language for all participants, potentially introducing a language barrier that could affect the comprehension and interpretation of the survey questions. This language discrepancy might lead to variations in how questions are understood and answered, possibly impacting the accuracy and reliability of the responses received. To mitigate this issue and ensure a broader comprehension of the questionnaire, future iterations of this study could benefit from providing translations of the survey into Arabic, the native language of Saudi Arabia.

Lastly, it is crucial to acknowledge the use of social media for data collection carries inherent limitations that may impact the validity and generalizability of the findings. While these platforms enabled wider reach, they were susceptible to introducing selection bias. This bias stems from the fact that access to social media is not uniform across all demographics, leading to potential underrepresentation of certain groups who may have limited internet access, lower technology literacy, or simply prefer not to use social media.

## Conclusions

Our findings suggest that the study participants' knowledge, attitude, and practice related to CSS use are inadequate, although they are reflected more in the capital regions. CSS for children is underused despite parents being aware of its availability and value. Media and policymakers must collaborate to improve these using tailored interventions. Based on the knowledge gap suggested in the present study, awareness programs must incorporate detailed instructions and guidelines for proper CSS use. Conducting further research to explore the correlation between government-imposed penalties and child safety seat usage could provide a more accurate understanding of the situation.

## Appendices

This questionnaire will be used for research purpose only.

Do you agree to participate:                   yes                 No

Did you fill this research before:            yes                 No

Demographic Data

Gender:                             Male                                        Female

Age (years)

Education:                        Illiterate                                   Primary / secondary       

                                           High school / Diploma           Bachelor and above

Number of children

Age intervals of participants’ children (years):

         Less than 2                                  2-4                                  5-8                             more than 8

Family income (SAR):                 Below 4,000                        4,000-7,000 

                                            8,000-10,000                    >10,000

Other drivers:                 

          Siblings                                Grandparents                       Driver                              Others    

Are they use the car safety seat?    

           Yes                                        No

Knowledge

Do you know about car safety seats?                    

Yes                            No

If yes,

How did you hear about car safety seats?

1.      Independently researched

2.      I saw someone with a car safety seat

3.      TV, media, journal, advertisement

4.      Advice from doctor/nurse

5.      Other

Where is the best place for a child to sit in a car?

1.      Back seat                       

2.      Front seat                            

3.      No difference

Which way should your child face in a safety seat in the car?

1.      Should always face the rear of the car

2.      Should always face the front of the car

3.      Should face the rear of the car until they are at least 1 year of age

Should we change the car safety seat according to the age of the baby?                 

Yes                          No

Are you aware of laws in Saudi Arabia against traveling with unrestrained children?

Yes                               No

Do you think that we need more education for the community regarding car safety seats?    

Yes                               No

Attitude

Are you using seat belt (seat belt use for parents)          

Yes                             No

Have you ever used care safety seats?           

Always                      sometimes                                 rare                                   never

If No, why?

Expensive                          crowded (no space)              Not essential device

Other ………………………………………………….

Have you ever had a car accident with your child in the car?

Yes                             No

If yes, were you using the child safety seat?

Yes                            No

What was the outcomes of that acceding according to the child situation:

Death        severely affected       mild affected       no effect 

Answer by using scale from strongly agree to strongly disagree the following questions:

-          Believed that an adult carrying a child with or without restraint is acceptable

1.    Strongly Agree

2.    Agree

3.    Undecided

4.    Disagree

5.    Strongly Disagree

-          A child occupying the front seat of the car is appropriate if he/she wears a seat belt.

1.    Strongly Agree

2.    Agree

3.    Undecided

4.    Disagree

5.    Strongly Disagree

-         Children ≥8 years no longer needed a booster seat once the car seat belt fits them properly

1.    Strongly Agree

2.    Agree

3.    Undecided

4.    Disagree

5.    Strongly Disagree

-          Car safety seat is essential device while driving with

1.    Strongly Agree

2.    Agree

3.    Undecided

4.    Disagree

5.    Strongly Disagree

-          Car safety seat is only important when driving fast/ long distance   

1.    Strongly Agree

2.    Agree

3.    Undecided

4.    Disagree

5.    Strongly Disagree

-          Had been cautioned at least once for non-use of CCR for their children while driving

1.    Strongly Agree

2.    Agree

3.    Undecided

4.    Disagree

5.    Strongly Disagree
